# Creatine supplementation and respiratory muscle function in health and disease: translational insights from human to mouse

**DOI:** 10.1183/23120541.00305-2026

**Published:** 2026-09-14

**Authors:** Oliver J. Price, James Brown, Emily Storey, Louise Turner, Michael Johnson, Graham Sharpe, Stephen B. Wheatcroft, Nadira Yuldasheva, Paul W. Hendrickse, Marcelo G. Pereira, T. Scott Bowen

**Affiliations:** 1 School of Biomedical Sciences, Faculty of Biological Sciences, University of Leeds, UK; 2 Department of Respiratory Medicine, Leeds Teaching Hospitals NHS Trust, Leeds, UK; 3 School of Education, Health and Science University of Gloucestershire, Gloucester, UK; 4 Department of Sports Science, School of Science and Technology, Nottingham Trent University, Nottingham, UK; 5 Leeds Institute of Cardiovascular and Metabolic Medicine, University of Leeds, Leeds, UK; 6 Lancaster Medical School, Lancaster University, Lancaster, UK; 7 P. Hendrickse and M. Pereira contributed equally to this article; 8 O.J. Price and T.S. Bowen contributed equally to this article and lead authors and supervised the work

## Abstract

**Background:**

Respiratory muscle weakness contributes to exercise intolerance in both ageing and disease; however current treatment options remain limited. In this study, we investigated the potential for creatine (Cr) supplementation to increase inspiratory muscle strength (PImax) in health and mitigate diaphragm dysfunction in disease.

**Methods:**

Using a translational approach, we combined a human inspiratory muscle training (IMT) intervention with a mechanistic preclinical heart failure (HF) model. 29 healthy males (forced expiratory volume in 1 s >80% pred) completed 4 weeks of IMT (30 breaths, twice daily, at 50% PImax), either alone (IMT, n=15) or with Cr (IMT+Cr, n=14). PImax and expiratory muscle strength (PEmax) were assessed pre- and post-IMT. Subsequently, isolated diaphragm fibre bundles from healthy and HF mice underwent *in vitro* analysis following 3 weeks of Cr supplementation or placebo.

**Results:**

In humans, baseline PImax was similar between groups (p=0.554). The 33% increase in PImax after IMT+Cr was greater than the 18% increase after IMT (condition×time interaction; p=0.031). In mice, Cr had no effect in health but increased diaphragm function in HF with improvements in specific force, twitch relaxation, and fatigue resistance (all p<0.05).

**Conclusion:**

Cr supplementation was associated with a greater increase in PImax following IMT in healthy adults and improved diaphragm contractile performance in a preclinical model of HF. These findings provide biological plausibility for a potential effect of Cr on intrinsic diaphragm function and warrant confirmation in adequately powered, randomised controlled trials.

## Shareable abstract

**Creatine supplementation is associated with greater increases in *P*_Imax_ following IMT in healthy humans and improved diaphragm contractile performance in a preclinical model of heart failure** https://bit.ly/4cxAaid

## Introduction

Respiratory muscle weakness, defined as a reduced force-generating capacity and/or contractile velocity of the diaphragm, is a recognised feature of advanced neuromuscular and neurological disorders [1, 2]. It is also commonly observed with advancing age and in chronic disease states such as COPD and heart failure (HF) [3, 4]. In COPD, airflow limitation and dynamic hyperinflation increase the work of breathing and place the inspiratory muscles at a mechanical disadvantage, impairing contractile efficiency [5, 6]. In contrast, HF is characterised by diaphragm myopathy and intrinsic contractile dysfunction, which contribute to dyspnoea and exercise intolerance [7].

Inspiratory muscle training (IMT) *via* pressure-threshold loading is an established intervention that improves inspiratory muscle strength, fatigue resistance, exercise tolerance and perceptions of dyspnoea in both health and disease [8, 9]. The increase in inspiratory muscle strength following IMT is attributed to both neural and structural adaptations, including improved motor-unit recruitment [10] and hypertrophy of the diaphragm [11] and external intercostals [12], which closely mimics locomotor muscle adaptation following resistance training [13]. Importantly, combining resistance training with exogenous creatine (Cr) supplementation enhances intracellular phosphocreatine (PCr) availability and ATP resynthesis, which enable higher-intensity training sessions to increase peripheral muscle adaptations [14]. However, the effect of exogenous Cr on respiratory muscle function in both health and disease has yet to be explored [15].

While Cr primarily acts as a metabolic buffer, several pro-anabolic and anti-catabolic mechanisms in peripheral locomotor muscles have also been proposed in healthy individuals, including upregulation of myosin heavy chain and myogenic regulatory factor expression, enhanced satellite-cell proliferation and differentiation, increased myonuclear content, and elevated insulin-like growth factor (IGF)-1 levels [16–19]. Cr has also been shown to attenuate the rise in serum myostatin (a negative regulator of muscle growth) during resistance training in healthy humans [20]. Collectively, these findings highlight that Cr may exert direct effects on muscle function both independently and in response to exercise training.

In the context of disease, Cr has been shown to increase fat-free mass and peripheral muscle strength when used alongside exercise-based rehabilitation in patients with COPD [21] or HF [22, 23]. However, many patients are unable or unwilling to participate in structured exercise-based interventions and may experience chronically elevated respiratory muscle loading due to persistent hyperventilation. This sustained ventilatory demand represents a metabolic and mechanical stress on the inspiratory muscles and may contribute to diaphragm dysfunction in susceptible individuals [7]. As such, in conditions such as HF, Cr supplementation may represent a potential adjunct strategy to support respiratory muscle bioenergetics and thus mitigate diaphragm dysfunction.

While some studies in HF have reported statistical and clinically meaningful benefit when combining Cr supplementation with exercise training [22, 23], others have failed to demonstrate an effect [24, 25]. The disparity in findings between studies may relate to differences in either treatment duration and/or dose, as well as methods of functional assessment. In humans, quantifying respiratory muscle strength often relies on indirect, volitional measures such as maximal inspiratory and expiratory mouth pressures [26]. However, the most direct assessment of contractile function requires isolating diaphragm muscle fibres, typically obtained *via* invasive biopsy during medically indicated surgery [27]. To overcome these constraints and provide a direct evaluation of the effects of Cr supplementation on respiratory muscle function, experimental animal models offer a viable alternative that enable mechanistic investigation at the fibre level and provide a window to explore therapeutic effects.

This study therefore adopted a translational approach by combining human assessments of inspiratory muscle strength with preclinical mechanistic experiments in mice to explore the effects of Cr supplementation on respiratory muscle function across health and disease. The study aims were two-fold: 1) investigate the effects of Cr supplementation in combination with IMT on PImax in healthy humans; 2) investigate the effects of Cr supplementation on mouse diaphragm contractile function in health and disease, specifically testing the ability to attenuate HF-induced diaphragm weakness. We hypothesised that oral Cr supplementation combined with IMT would enhance PImax in healthy individuals in comparison to IMT alone and attenuate diaphragm contractile dysfunction in mice with disease.

## Methods

This study was approved by the local research ethics committee, and all participants provided written informed consent. All animal procedures complied with the UK Animals (Scientific Procedures) Act 1986 and were approved by the University of Leeds Animal Welfare and Ethical Review Committee. Full experimental protocols are provided in the supplementary methods.

### Aim 1: human experimental design and procedures

Thirty recreationally active males (age 21±1 year; height 184±8 cm; body mass 78±11 kg; BMI 23±3 kg·m^−2^) with normal resting lung function (forced expiratory volume in 1 s (FEV_1_) >80% predicted) were recruited. Participants were nonsmokers, asymptomatic, free from chronic disease and not taking regular medication. Habitual diet and activity were maintained, and exercise, alcohol and caffeine were avoided for 24 h before each visit. Testing was conducted 2 h post-prandially at a similar time of day (±1 h). Participants attended three visits: familiarisation, baseline assessment and post-intervention. Testing at each visit included pulmonary function and respiratory muscle strength assessments. Participants were matched for baseline maximal inspiratory pressure (PImax) and allocated to IMT or IMT combined with creatine (Cr) supplementation. Creatine monohydrate (>98% purity) was administered orally (20 g·day^−1^ for 7 days followed by 3 g·day^−1^ thereafter). The intervention lasted 4 weeks, with IMT adherence self-reported using a home-based diary.

Pulmonary function was assessed using forced spirometry (Vyntus PNEUMO, Vyaire Medical, Germany). PImax and maximal expiratory pressure (PEmax) were measured using a handheld mouth pressure meter (MicroRPM, Micro Medical, UK). Measurements were repeated until the highest three values varied by <10%, with the highest value reported. IMT consisted of 30 consecutive dynamic inspiratory efforts, performed twice daily (∼12 h apart) for 4 weeks using a pressure-threshold device (POWERbreathe, Gaiam, UK). Training commenced at 50% of baseline PImax. Resistance was adjusted during the intervention to maintain a high-intensity training stimulus in accordance with principles of progressive overload.

### Aim 2: mouse experimental design and procedures

Male and female C57BL/6 mice (12 weeks of age) were allocated to healthy+placebo, healthy+Cr, HF+placebo or HF+Cr groups. Mice received standard drinking water or creatine monohydrate (>98% purity; at 30 mg·g^−1^·day^−1^) for 3 weeks, with investigators blinded to treatment. HF was induced by permanent ligation of the left anterior descending coronary artery, with myocardial infarction confirmed by echocardiography at 1 week. Mice were randomised to treatment 4 weeks post-surgery, and terminal experiments were performed 7 weeks post-surgery. Organs were dissected and weighed, infarct size quantified from Picrosirius-red-stained cardiac sections, and diaphragm contractile function assessed *in vitro* using established protocols.

### Statistical analysis

Data were analysed using GraphPad Prism (v.10.0). Normality was assessed using Shapiro–Wilk tests. Human data were analysed using independent t-tests and two-way repeated-measures ANOVA (condition×time). Mouse data were analysed using two- or three-way ANOVA. Where appropriate, *post hoc* comparisons were adjusted for multiple testing using Bonferroni correction, including across force frequency and fatigue protocols in the animal experiments. Data are presented as mean±sd, with statistical significance set at p<0.05. No formal *a priori* sample size calculation was performed, as the study was designed as an exploratory, proof-of-concept investigation informed by feasibility and prior work using comparable protocols.

## Results

### Aim 1: effect of creatine on inspiratory muscle strength in healthy humans

29 participants completed the study. One individual in the IMT+Cr group was excluded for not achieving ≥80% adherence to the IMT protocol. All remaining participants demonstrated high adherence: 95±3% and 96±2% in the IMT and IMT+Cr groups, respectively. Self-reported adherence to Cr supplementation was also high (98±1%).

Baseline pulmonary function was not different between groups (p>0.05). For FEV_1_ and forced vital capacity (FVC), there were no significant condition×time interactions (p=0.119 and p=0.180, respectively). A main effect of time was observed for FEV_1_ (p=0.041), reflecting an overall increase across the interventions, whereas no main effect of condition was detected (p=0.984). In contrast, FEV_1_/FVC demonstrated a significant condition×time interaction (p=0.020), driven by an increase in the IMT+Cr group compared with no change following IMT alone. For peak inspiratory flow (PIF) and peak expiratory flow, no significant condition×time interactions were observed (p>0.05); however, a main effect of time was detected for PIF (p=0.007), indicating an overall increase across both groups (table 1).

**TABLE 1:** Pulmonary function pre- and post 4-week IMT intervention

	IMT (n=15)		IMT+Cr (n=14)		p-value		
	Pre	Post	Pre	Post	Time	Condition	Interaction
**FEV_1_, L**	4.56±0.75 (97±9%)	4.57±0.73	4.51±0.74 (101±13%)	4.60±0.68	0.041	0.984	0.119
**FVC, L**	5.40±0.98 (100±9%)	5.42±0.96	5.62±0.71 (95±17%)	5.58±0.69	0.591	0.557	0.180
**FEV_1_/FVC, %**	88±8 (112±6%)	88±8	89±8 (114±5%)	91±8	0.133	0.542	0.020*
**PEF, L·min^−1^**	583±123	591±72	559±153	583±125	0.204	0.188	0.091
**PIF, L·min^−1^**	464±97	503±93	469±154	505±117	0.007	0.934	0.913

Data are presented as mean±sd. IMT: inspiratory muscle training; Cr: creatine; FEV_1_: forced expiratory volume in 1 s; FVC: forced vital capacity; PEF: peak expiratory flow; PIF: peak inspiratory flow. *: p<0.05.

**FIGURE 1:**
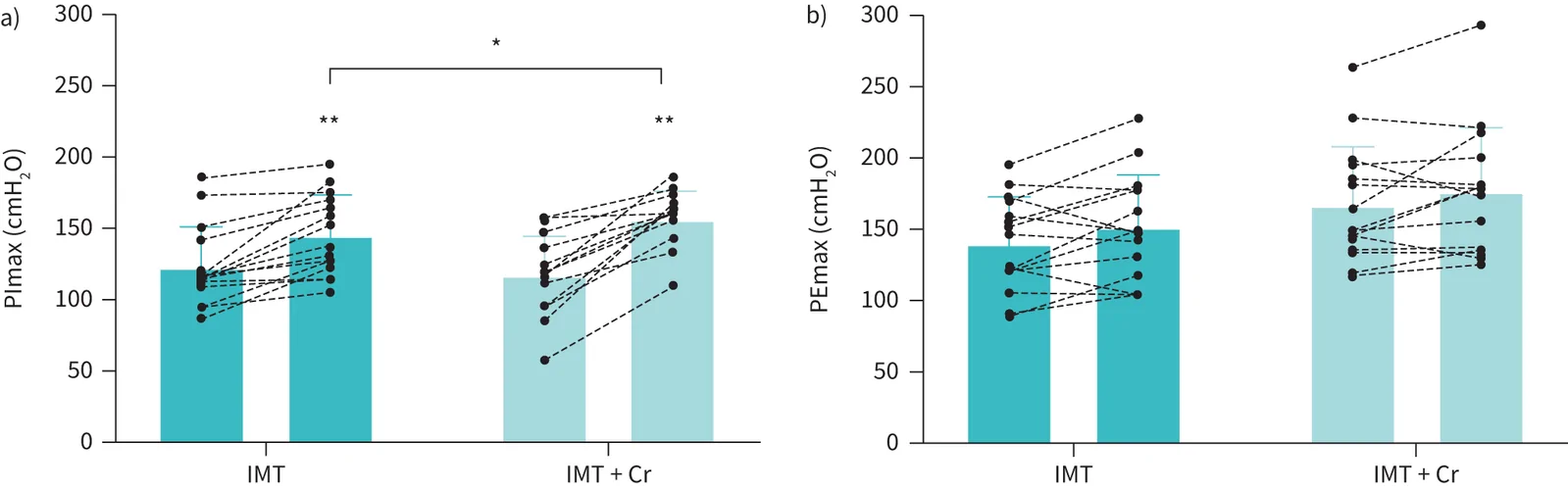
a) Maximal inspiratory pressure (PImax) and b) maximal expiratory pressure (PEmax) before and after 4 weeks of inspiratory muscle training (IMT) or IMT plus creatine supplementation (Cr). Data are shown as individual responses with mean±sd. **: p<0.001 for within-group pre-post changes. *: p<0.05 for condition×time interaction. The post-intervention annotation denotes the between-condition difference in the magnitude of change corresponding to this interaction.

Baseline PImax was not different between groups (p=0.554). For PImax, there was a significant condition×time interaction (p=0.031), with the increase in PImax being greater after IMT+Cr (117±28 to 156±20 cmH_2_O, +33%; p<0.001) compared with IMT alone (123±28 to 145±28 cmH_2_O, +18%; p<0.001) (figure 1a). The between-group difference in change in PImax was 17 cmH_2_O (95% CI 2–32), corresponding to a moderate-to-large effect size (Cohen's d=0.8). Baseline PEmax was not different between groups (p=0.081), and there was no significant condition×time interaction (p=0.868); however, a main effect of time was detected (p=0.011), indicating an overall improvement across both groups (figure 1b).

### Aim 2: effect of creatine on mice diaphragm contractile function in health and disease

**FIGURE 2:**
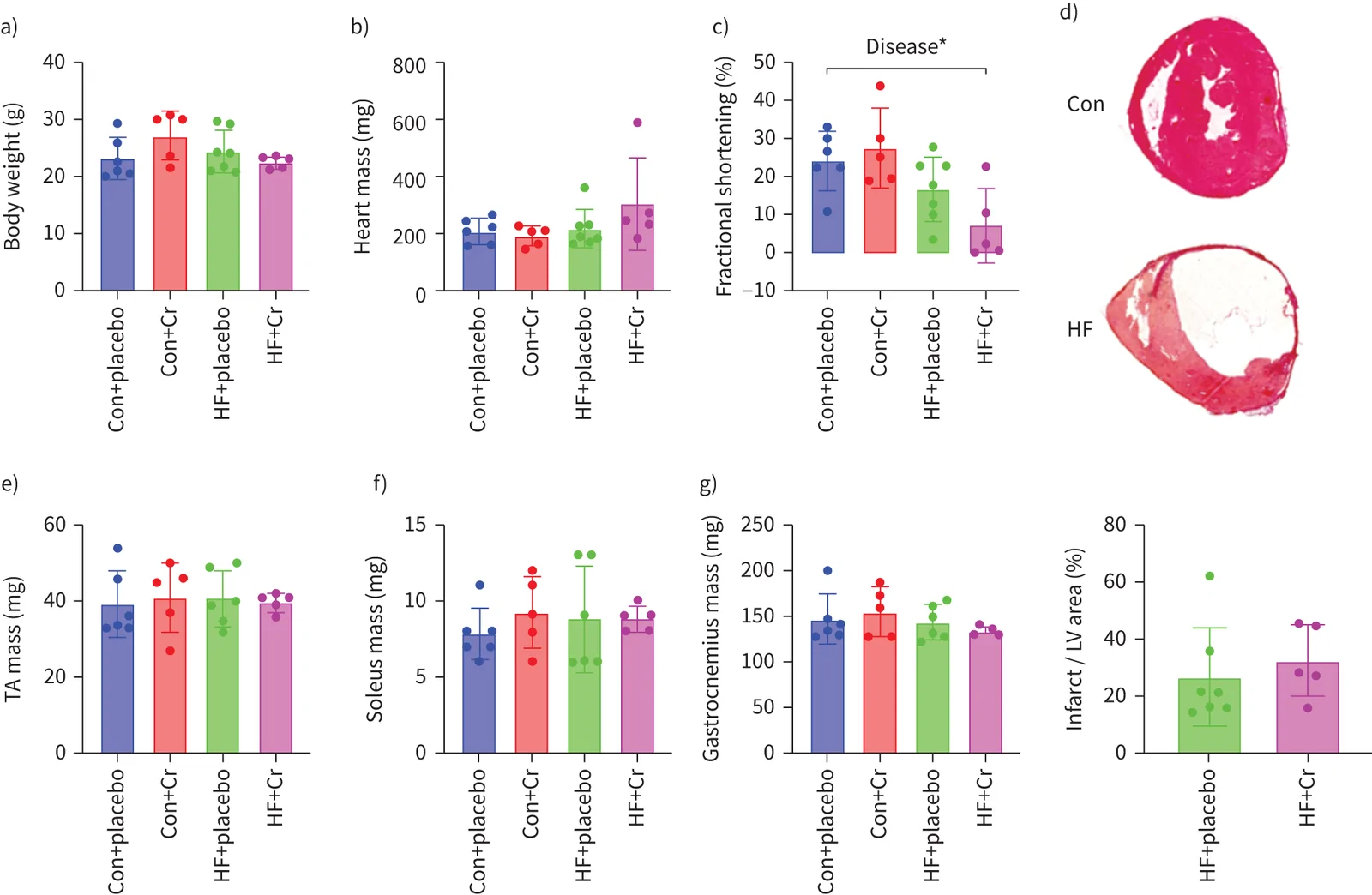
Physical, cardiac, and skeletal muscle assessments in healthy control (Con) and heart failure (HF) mice (following 4 weeks of myocardial infarction (MI)), supplemented thereafter with creatine (Cr) or placebo for 3 weeks. a) Body mass was assessed, along with cardiac indices of b) mass, c) function (echocardiography) and d) remodelling (representative stained Sirius red-stained sections above, with quantified infarct size below). e–g) Wet mass of hindlimb skeletal muscles, including tibialis anterior (TA), soleus and gastrocnemius. LV: left ventricle. *: Main effect of disease, p<0.05.

No condition×disease interaction (p>0.05) or main effect of condition (Cr supplementation; p>0.05) was observed for cardiac function, body weight, heart mass or skeletal muscle wet masses across the tibialis anterior, soleus or gastrocnemius muscles (all p>0.05; figure 2a–g). However, there was a significant main effect of disease on cardiac function (p=0.015), such that mice with HF demonstrated ventricular dysfunction (figure 2c) and pathological remodelling (figure 2d) compared with healthy controls.

**FIGURE 3:**
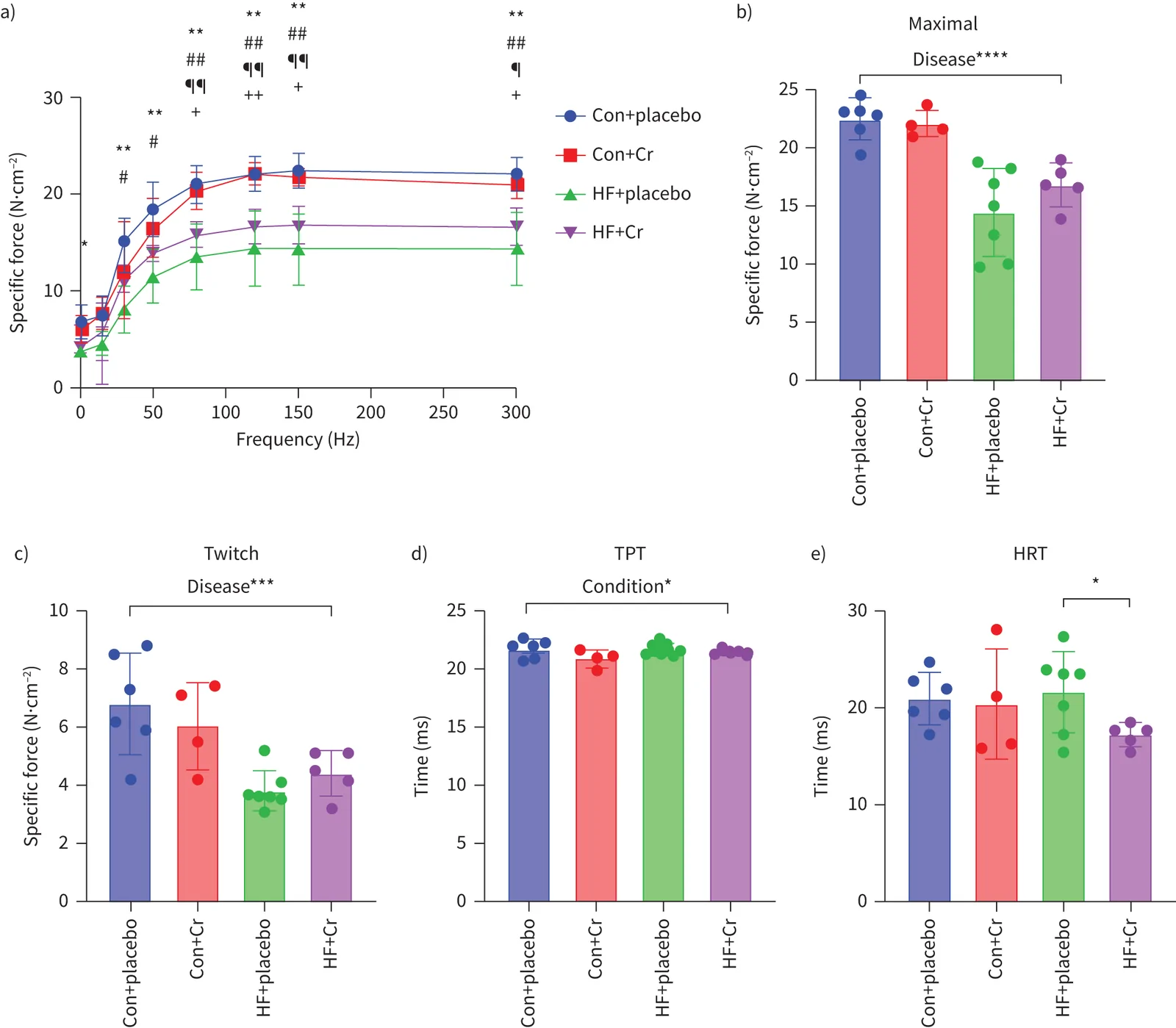
Direct assessment of *in vitro* contractile function of isolated diaphragm fibre bundles from healthy control (Con) and heart failure (HF) mice supplemented with creatine (Cr) or placebo for 3 weeks. a) Diaphragm-specific force–frequency relationship, b) maximal tetanic force, c) twitch force and d) kinetics (time to peak tension (TPT) and e) half-relaxation time (HRT)) were assessed. Interactions denoted in text: main effect (condition), main effect (disease). a) significant between-group differences are shown as *: Con+placebo *versus* HF+placebo. ^#^: Con+placebo *versus* HF+Cr. ^¶^: Con+Cr *versus* HF+placebo. ^+^: Con+Cr *versus* HF+Cr. One symbol indicates p<0.05, two identical symbols indicate p<0.01. ***: p<0.001. ****: p<0.0001.

**FIGURE 4:**
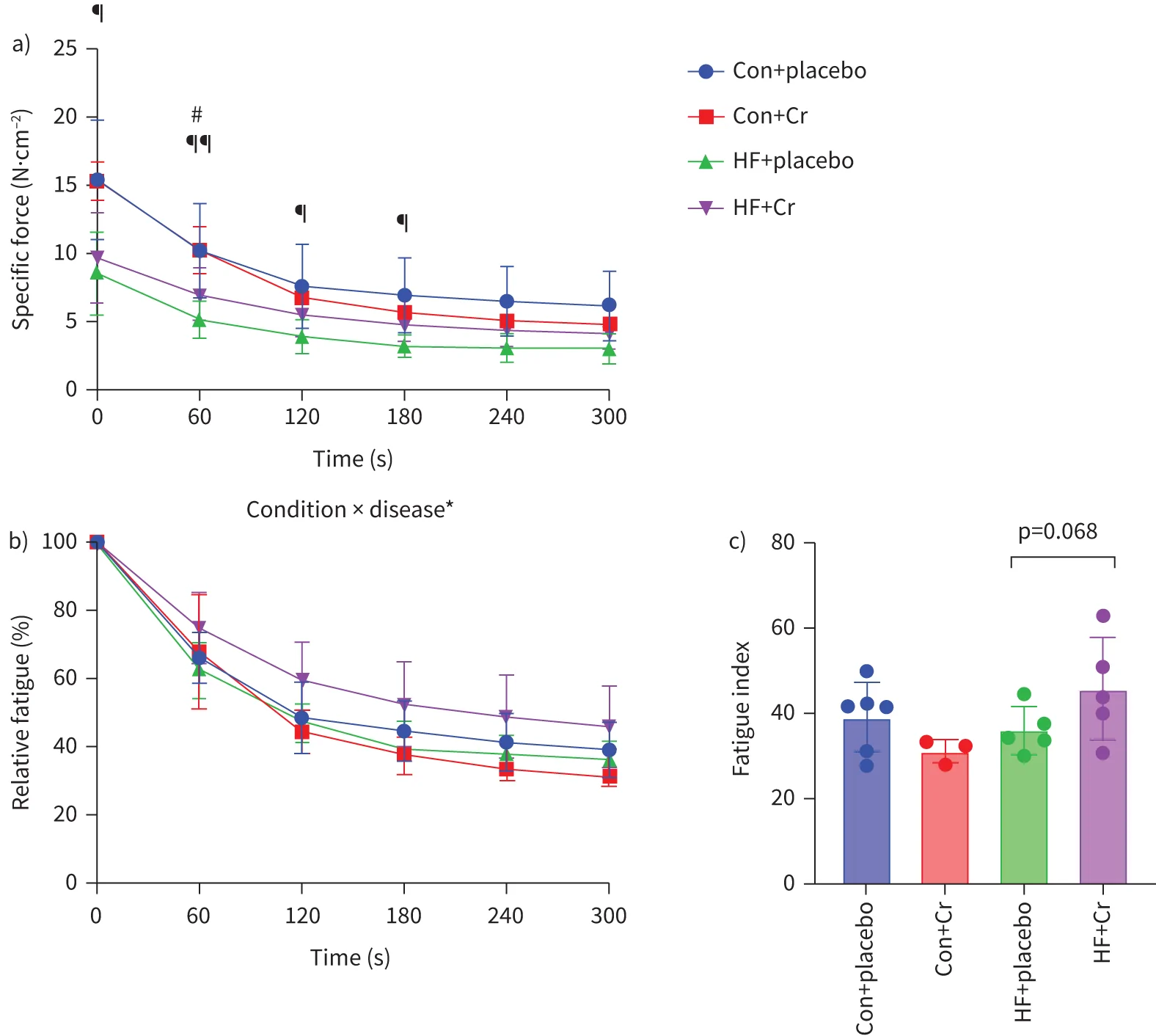
*In vitro* diaphragm fatigue was assessed in isolated diaphragm fibre bundles from healthy control (Con) and heart failure (HF) mice supplemented with creatine (Cr) or placebo for 3 weeks. Diaphragm fibres were subjected to 5 min of repeated tetanic contractions (40 Hz), with measurements of a) specific force (absolute fatigue) and b) normalised relative fatigue, with c) the fatigue index quantified. a) significant between-group differences are shown as ^#^: Con+placebo *versus* HF+placebo. ^¶^: Con+Cr *versus* HF+placebo. One symbol indicates p<0.05, two identical symbols indicate p<0.01; b) *: p<0.05, condition×disease interaction.

In healthy control mice, direct stimulation of isolated diaphragm fibre bundles revealed no significant effect of Cr supplementation on diaphragm contractile function or fatigue properties (figures 3 and 4; all p>0.05), apart from decreasing rise time in twitch kinetics (figure 3d; p=0.049). However, as anticipated, HF induced diaphragm contractile weakness, decreasing specific forces across all force frequencies by approximately 30–40% (figure 3a; p<0.001). Specifically, twitch, submaximal and maximal contractile forces were reduced in HF mice compared with healthy controls (figure 3a–c; all p<0.001), although twitch kinetics remained unaltered (figure 3d–e; p>0.05). Moreover, the diaphragm showed greater absolute fatigue in mice with HF compared with healthy controls, generating lower forces in response to repeated stimulations (figure 4a; p=0.014), but relative fatigue was unchanged when quantified as the fatigue index (figure 4b; p=0.155).

HF+Cr was associated with partial attenuation of diaphragm weakness and fatigue. For example, although no significant condition×disease interaction was observed (p=0.075); exploratory *post hoc* comparisons demonstrated greater diaphragm force at 30 Hz (p=0.030) and 50 Hz (p=0.043) in HF+Cr compared with HF+placebo (figure 3a). There was no frequency×condition×disease interaction across the force-frequency relationship (p=0.728). Despite no condition×disease interaction (p=0.370), diaphragm twitch half-relaxation time was shorter in HF+Cr compared with HF+placebo (p=0.024; figure 3e). HF+Cr showed no effect on absolute diaphragm fatigue-time relationship (condition×disease interaction; p=0.295; figure 4a), however there was an interaction for relative diaphragm fatigue-time relationship (condition×disease; p=0.041; figure 4b). Although the fatigue index in the diaphragm was numerically higher in HF+Cr, this difference did not reach statistical significance (p=0.068; figure 4c). Collectively, however, these findings should be interpreted as exploratory and hypothesis-generating given the absence of a consistent condition×disease interaction across outcomes.

## Discussion

This translational study presents novel data on the effects of Cr supplementation on respiratory muscle function in health and disease. In healthy humans, Cr supplementation was associated with a greater increase in PImax following IMT, with a significantly greater increase (+17 cmH_2_O) in PImax in comparison with IMT alone. Based upon our findings indicating the potential for Cr supplementation to improve PImax, we subsequently utilised a unique approach that allowed us to directly explore contractile function in isolated diaphragm fibres using a preclinical mouse model of HF characterised by diaphragm myopathy. While Cr supplementation in healthy mice did not influence diaphragm function, it partially rescued diaphragm dysfunction in mice with HF. Collectively, therefore, these data suggest that Cr supplementation may represent a potential adjunct strategy for improving respiratory muscle function in health and disease. Importantly, the preclinical findings in the present study should be interpreted as providing biological plausibility and mechanistic context in disease, rather than direct mechanistic confirmation of the human results.

### Creatine is associated with greater IMT-induced increases in PImax in healthy humans

This is the first study in humans to assess the impact of IMT in conjunction with Cr supplementation on respiratory muscle adaptations. Our data show that IMT alone increased PImax by 18%, which corresponds to the magnitude of change previously observed in studies employing comparable IMT interventions [28]. Importantly, the addition of Cr alongside IMT resulted in a greater improvement in PImax from baseline (+33%), indicating an ergogenic effect of supplementation. The IMT protocol employed in the present study included passive unloaded expiration. While a significant main effect of time was observed for PEmax post-IMT, supporting an interaction between inspiratory and expiratory muscles (akin to agonist-antagonist muscle dynamics [29]), the addition of Cr conferred no further benefit to PEmax.

The increase in PImax following IMT likely reflects improved inspiratory muscle performance; however, no direct assessment of structural adaptation was performed in the present study. While previous studies have demonstrated structural remodelling of the diaphragm [11] and external intercostals [12]; the present study did not assess diaphragm morphology, and therefore structural adaptation cannot be inferred from the observed changes in PImax. Mechanistically, Cr supplementation supports the immediate muscle bioenergetic system, which predominates during vigorous-intensity, short-duration activity [30]. In skeletal muscle, Cr is rapidly and reversibly phosphorylated by creatine kinase to form PCr, which serves to buffer ATP concentrations during periods of physiological stress. Cr supplementation increases the intracellular pool of total Cr (Cr+PCr), enhancing ATP resynthesis and delaying PCr depletion during repeated effort [14]. Beyond metabolic buffering capacity, Cr has also been shown to be associated with adaptive remodelling of skeletal muscle, including higher myosin heavy chain and myogenic regulatory factor expression, satellite-cell proliferation and differentiation, myonuclear content and IGF-1 levels [16–19], alongside attenuating the rise in serum myostatin during resistance training [20]. While speculative, these collective mechanisms may extend to the diaphragm and accessory inspiratory muscles engaged during IMT, *via* pathways analogous to those described in limb skeletal muscle in response to resistance training [13].

### Creatine attenuates diaphragm contractile dysfunction in heart failure

Diaphragm myopathy is observed in a range of pathological conditions, including critical illness, sepsis and chronic diseases such as COPD, cancer, and HF [31]. In contrast, healthy ageing is associated with nonpathological remodelling of the diaphragm, which may contribute to functional decline [27]. In HF, diaphragm dysfunction is a well-recognised feature, closely associated with reduced exercise tolerance, increased symptom burden [7], and early mortality [32]. While diaphragm dysfunction has been identified as a potential therapeutic target [33], treatments have yet to be established. Cr supplementation has previously been shown to improve limb muscle strength, endurance and body composition in patients with HF following short-term high-dose regimens (*e.g.* 20 g·day^−1^ for up to 6 weeks) [22, 23], with similar benefits observed in older adults when combined with resistance training [34]. Despite this, the effects of Cr supplementation on diaphragm dysfunction in HF remain poorly characterised. In the present study, we provide preliminary evidence that Cr supplementation may improve diaphragm contractile performance in a mouse model of HF. In line with previous studies [35] and our human PEmax data, Cr supplementation had no effect on basal diaphragm function in healthy untrained mice, supporting the premise that its efficacy is contingent upon the presence of physiological or pathological stress (*i.e.* exercise or disease).

Importantly, our findings suggest that Cr supplementation may preserve aspects of diaphragm contractile performance in HF. As our functional data were collected from isolated diaphragm fibre preparations that were directly stimulated *in vitro* (hence overcoming any potential limitations associated with neural, vascular, or volitional mechanisms) with contractile data expressed as specific force to account for any differences in muscle mass, our data are consistent with potential improvements in intrinsic fibre function in the context of HF. The attenuation of diaphragm dysfunction may relate to improved excitation-contraction coupling transmission, which is known to be impaired in HF [27]. While our findings suggest improved force generation at the level of the myofilaments [36], we also observed improvements in twitch kinetics and submaximal force production, consistent with enhanced cytosolic calcium handling. This aligns with previous reports of Cr-mediated improvements in calcium homeostasis in skeletal muscle in the context of Duchenne muscular dystrophy [37]. Improved calcium handling likely contributed to greater fatigue resistance, as PCr depletion during fatigue impairs calcium reuptake [38], delaying muscle relaxation and suppressing force generation in subsequent contractions [39]. In addition to replenishing PCr stores and supporting ATP resynthesis, other potential mechanisms may include the potential to reduce inflammation, reactive oxygen species and catabolic signalling [40], which require further investigation.

### Methodological limitations and future research

Several limitations warrant consideration. First, no *a priori* sample size calculation was performed for either the human or preclinical experiments, and both components should therefore be interpreted as exploratory. The modest sample sizes increase the risk of both type I and type II error, particularly given the reliance on interaction effects in the human and animal experiments. Accordingly, the findings should be viewed as hypothesis-generating and require confirmation in adequately powered, randomised controlled trials.

Second, the human intervention did not include a placebo control for creatine supplementation, and neither participants nor investigators were blinded. As PImax is a volitional, effort-dependent outcome, the observed between-group differences may partly reflect motivational, expectancy or learning effects rather than a direct physiological effect of creatine; consequently, the human data should be interpreted as associative rather than causal. The human findings, therefore, cannot establish a causal physiological effect of creatine or rule out potential behavioural influences. In addition, PImax should be viewed in the context that is reflects global inspiratory pressure generation rather than diaphragm-specific contractile function and does not provide a measure of structural changes (*e.g.* diaphragm thickness or muscle morphology). Participants were stratified by baseline PImax and allocated to groups; however, no formal randomisation was performed, which may introduce allocation bias. No direct confirmation of creatine uptake (*e.g.* plasma or muscle creatine levels) was also performed, limiting mechanistic interpretation of the supplementation effects.

Third, the human cohort included a modest sample size, comprising healthy males only, and the intervention duration was relatively short, limiting generalisability to clinical populations. Although IMT was implemented pragmatically, the absence of detailed quantitative progression data (including reassessment frequency and achieved training intensities) limits the ability to fully characterise the training stimulus; therefore, potential between-group differences in training stimulus cannot be excluded. Initiating training breaths from functional residual capacity may have resulted in a relatively greater training intensity than 50% PImax referenced to residual volume. Accordingly, the relatively high-intensity IMT protocol employed in healthy individuals may limit direct extrapolation to clinical populations, in whom tolerance and feasibility may differ.

In the preclinical experiments, the study was not powered to assess sex-specific effects despite inclusion of both sexes, and the experimental deign examined disease-related diaphragm dysfunction rather than training-induced adaptation. Accordingly, the animal data provide biological plausibility and mechanistic context rather than direct confirmation of the human findings. Finally, clinical implications should be interpreted cautiously, as no patient data were collected.

### Conclusion

In summary, this exploratory translational study suggests that Cr supplementation is associated with greater increases in PImax following IMT in healthy humans and improved diaphragm contractile performance in a preclinical model of HF. The preclinical findings provide biological plausibility for a potential effect of creatine on intrinsic diaphragm contractility in disease. Further adequately powered, randomised controlled trials are required to confirm these findings and determine their clinical relevance.

## Supplementary material

**Please note:** supplementary material is not edited by the Editorial Office, and is uploaded as it has been supplied by the author. Supplementary material 00305-2026.SUPPLEMENT
