# Supplementary material for: Creatine supplementation and respiratory muscle function in health and disease: translational insights from human to mouse
Source: ERJ Open Res. 2026 Sep 14;12(5):00305-2026. doi: 10.1183/23120541.00305-2026 (PMC13573218; doi:10.1183/23120541.00305-2026)
Supplement: Supplementary file 1 [file 00305-2026.SUPPLEMENT.pdf]

SUPPLEMENTARY MATERIAL

Methods

Aim 1: Human experimental design and procedures

Thirty recreationally active males (age: 21 ± 1 yr; height: 184 ± 8cm; body mass: 78 ± 11kg; BMI: 23 ± 3kg/m^2) with normal resting lung function (FEV_1 >80% pred) were recruited to participate. All were non-smokers, entirely asymptomatic, free from chronic disease and not taking any regular medication. Participants were instructed to maintain their usual diet and activity level throughout the study and were instructed to abstain from exercise, alcohol, and caffeine 24 h prior to each visit. To minimise diurnal variation, all testing was conducted 2 h postprandially and at a consistent time of day (±1 hr). Participants attended the laboratory on three occasions. Visit 1 comprised familiarisation with all testing procedures. Visit 2 comprised baseline pulmonary function and respiratory muscle strength assessments. Participants were stratified by baseline PImax and assigned to either IMT alone or IMT combined with Cr supplementation (IMT + Cr) to achieve comparable baseline values; formal randomisation was not performed. Cr monohydrate (>98% purity) was dissolved in water and administered at a dose of 20 g·day^{-1} for 7 days (loading phase), followed by 3 g·day^{-1} for the remainder of the intervention (maintenance phase) [1]. The intervention lasted 4 weeks and IMT adherence was self-reported in a home-based diary. After the intervention, visit 3 comprised a repeat of pulmonary function and respiratory muscle strength assessments.

Pulmonary function and respiratory muscle strength measurements

Forced spirometry was performed using a Vyntus PNEUMO spirometer (Vyaire Medical, Leibnistrasse, Höchberg, Germany) [2]. PImax and maximal expiratory muscle pressure (PEmax) were assessed using a hand-held mouth pressure meter (MicroRPM, Micro Medical Ltd., Kent, UK) to provide indices of global inspiratory and expiratory muscle strength. PImax and PEmax (assessed from residual volume and total lung capacity, respectively) were recorded at 30-second intervals

until the variability of the highest three values was less than 10%, with the highest value reported [3].

Inspiratory muscle training intervention

Participants completed 30-consecutive dynamic inspiratory efforts initiated from functional residual capacity (relaxed end-expiration), twice daily (separated by ~12 h) using an inspiratory pressure-threshold loading device (POWERbreathe®, Gaiam, UK) over a 4-week period. Training was performed at an initial load equivalent to 50% PImax [4]. The resistance was progressively increased by the participants throughout the intervention to ensure participants could complete 30 satisfactory efforts per session, thereby maintaining an appropriate training stimulus through progressive overload.

Aim 2: Mouse experimental design and procedures

Four groups of C57/BL6 male and female mice aged 12 weeks were studied, including healthy placebo (n=6), healthy + Cr (n=5), HF + placebo (n=7), and HF + Cr (n=5). Mice received placebo (standard drinking water) or 3 weeks of Cr monohydrate (>98% purity; at 30 $mg \cdot g^{-1} \cdot day^{-1}$ via drinking water, consistent with past studies in mice to maximise muscle Cr / PCr concentrations and in line with standard human doses [5]. Investigators were blinded to treatment groups throughout the study.

Preclinical heart failure model

Mice in both HF + placebo and HF + Cr groups underwent established myocardial infarction surgery to induce systolic HF with a reduced ejection fraction, where a surgical silk suture ligated the left anterior descending coronary artery, as previously described [6-8]. Echocardiography was performed 1 week post-surgery to confirm induction of myocardial infarction following published methods [6-8].

Left ventricular end-diastolic diameter and end-systolic diameter were measured in M-mode to calculate fractional shortening. Mice were subsequently randomized to placebo or Cr supplementation 4 weeks post-surgery (i.e., timepoint when diaphragm dysfunction is established [7]). Terminal experiments were performed 7 weeks post-surgery, which included organs (heart and skeletal muscle from hindlimbs and diaphragm) being dissected and weighed, and the diaphragm prepared for functional analysis. Muscle mass of one mouse (HF + placebo group) was not recorded. Hearts were embedded in optimal cutting temperature compound, snap-frozen in liquid nitrogen-cooled isopentane, and stored at -80 °C. Transverse sections (10 μm) were cut at 20°C, mounted on polyline-coated slides, and stained (Picrosirius red) and imaged. Infarct size (%) was quantified via ImageJ software, as the ratio of infarct area to total left ventricular area [6-8].

Diaphragm contractile function

A fibre bundle was isolated from the costal diaphragm to assess *in vitro* contractile performance using a length-controlled lever system (Model 301B, Aurora Scientific Inc., Aurora, Canada), using our published protocols [27,28]. The muscle bundle was mounted vertically in a buffer-filled organ bath maintained at ~22°C and adjusted to optimal length (L_o). Following a 15-min equilibration period, isometric force was assessed via a force-frequency protocol ranging from 1 to 300 Hz (600 mA; 500 ms train duration; 0.25 ms pulse width). After a 5-min period in which muscle length was measured using a digital calliper, the muscle underwent a 5-min fatigue protocol (40 Hz every 2 s). The muscle was subsequently detached, trimmed free from rib and tendon, blotted dry on filter paper, and weighed. One sample from the Con + Cr group was excluded from all analysis due to failure to stimulate, whereas another sample from the Con + Cr group and two samples from the HF + placebo were excluded from the fatigue analysis due to a damaged preparation. Force (N) was normalised to muscle cross-sectional area (CSA; cm^2), calculated by dividing muscle mass (g) by the product of L_o (cm) and muscle density (assumed to be 1.06 $g \cdot cm^{-3}$), yielding specific force ($N \cdot cm^{-2}$).

Relative fatigue (%) was calculated by normalising force to the first contraction, with the fatigue index quantified as the ratio of end to initial force determined as a %.

Statistical analysis

Data were analysed using GraphPad Prism (version 10.0; GraphPad Software, San Diego, CA, USA). Normality was assessed using the Shapiro-Wilk test. For humans, independent samples t-tests were used to evaluate between group differences for PImax at baseline and a two-way repeated-measures ANOVA was used to evaluate pulmonary function and respiratory muscle strength outcomes in humans (condition x time). For mice, a two-way repeated-measures ANOVA was used to evaluate changes in cardiac and skeletal muscle properties in mice (condition x disease), whereas a three-way ANOVA was applied in diaphragm functional protocols as appropriate to account for frequency (condition x disease x frequency) or time (condition x disease x time). Where significant main or interaction effects were identified, Bonferroni-corrected paired t-tests were used for within-group comparisons (pre vs. post), and independent samples t-tests were used for between-group comparisons. For direct comparison on diaphragm function, an additional unpaired t-test was conducted to compare HF mice with and without Cr supplementation. Homogeneity of variance was assessed using Levene's test, and Greenhouse-Geisser corrections were applied where assumptions of sphericity were violated. Data are presented as mean ± SD, with statistical significance set at $P<0.05$.

References

1. Burke R, Piñero A, Coleman M, Mohan A, Sapuppo M, Augustin F, Aragon AA, Candow DG, Forbes SC, Swinton P, Schoenfeld BJ. The Effects of Creatine Supplementation Combined with Resistance Training on Regional Measures of Muscle Hypertrophy: A Systematic Review with Meta-Analysis. *Nutrients* 2023: 15(9).

2. Graham BL, Steenbruggen I, Miller MR, Barjaktarevic IZ, Cooper BG, Hall GL, Hallstrand TS, Kaminsky DA, McCarthy K, McCormack MC, Oropez CE, Rosenfeld M, Stanojevic S, Swanney MP, Thompson BR. Standardization of Spirometry 2019 Update. An Official American Thoracic Society and European Respiratory Society Technical Statement. *Am J Respir Crit Care Med* 2019: 200(8): e70-e88.

3. Laveneziana P, Albuquerque A, Aliverti A, Babb T, Barreiro E, Dres M, Dubé BP, Fauroux B, Gea J, Guenette JA, Hudson AL, Kabitz HJ, Laghi F, Langer D, Luo YM, Neder JA, O'Donnell D, Polkey MI, Rabinovich RA, Rossi A, Series F, Similowski T, Spengler CM, Vogiatzis I, Verges S. ERS statement on respiratory muscle testing at rest and during exercise. *Eur Respir J* 2019: 53(6).

4. Johnson MA, Sharpe GR, Brown PI. Inspiratory muscle training improves cycling time-trial performance and anaerobic work capacity but not critical power. *Eur J Appl Physiol* 2007: 101(6): 761-770.

5. Kurosawa Y, Degrauw TJ, Lindquist DM, Blanco VM, Pyne-Geithman GJ, Daikoku T, Chambers JB, Benoit SC, Clark JF. Cyclocreatine treatment improves cognition in mice with creatine transporter deficiency. *J Clin Invest* 2012: 122(8): 2837-2846.

6. Mangner N, Bowen TS, Werner S, Fischer T, Kullnick Y, Oberbach A, Linke A, Steil L, Schuler G, Adams V. Exercise Training Prevents Diaphragm Contractile Dysfunction in Heart Failure. *Med Sci Sports Exerc* 2016: 48(11): 2118-2124.

7. Bowen TS, Mangner N, Werner S, Glaser S, Kullnick Y, Schrepper A, Doenst T, Oberbach A, Linke A, Steil L, Schuler G, Adams V. Diaphragm muscle weakness in mice is early-onset post-myocardial infarction and associated with elevated protein oxidation. *J Appl Physiol (1985)* 2015: 118(1): 11-19.

8. Adams V, Bowen TS, Werner S, Barthel P, Amberger C, Konzer A, Graumann J, Sehr P, Lewis J, Provaznik J, Benes V, Büttner P, Gasch A, Mangner N, Witt CC, Labeit D, Linke A, Labeit S. Small-molecule-mediated chemical knock-down of MuRF1/MuRF2 and attenuation of diaphragm dysfunction in chronic heart failure. *J Cachexia Sarcopenia Muscle* 2019: 10(5): 1102-1115.
